# A warning against associating doxycycline with isotretinoin

**DOI:** 10.5935/0004-2749.20200074

**Published:** 2020

**Authors:** Alexandre Xavier da Costa, Gabriel Lima Benchimol, Tulio Frade Reis

**Affiliations:** 1 Department of Ophthalmology and Visual Sciences, Universidade Federal de São Paulo, São Paulo, SP, Brazil; 2 Universidade Estácio de Sá, Rio de Janeiro, RJ, Brazil; 3 Department of Ophthalmology, Otorhinolaryngology and Head and Neck Surgery, Universidade de São Paulo, Ribeirão Preto, SP, Brazil

Dear Editor:

After reading with great interest the article recently published by Andrade et al.^(1)^ about the use of isotretinoin and doxycycline to treat rosacea, we became concerned about an inadvertent possible treatment effect when using both drugs simultaneously.

The use of isotretinoin to treat severe forms of rosacea has been reported, but this medication can also worsen ocular symptoms as a result of dry eye, blepharitis, and hordeolum, as well described by Andrade et al.^(1)^.

Although the authors indicate that low-dose isotretinoin may be associated with a good treatment response in patients with rosacea along with reduced general side effects, worsening of ocular symptoms was notably pronounced in these patients, in contrast to what was observed in the doxycycline group. This might suggest to readers, especially dermatologists and ophthalmologists, that the use of doxycycline might be a good choice for patients suffering from blepharitis and rosacea combined with isotretinoin. Nonetheless, the authors did not mention that the association of both drugs is contraindicated because of the elevated risk of pseudotumor cerebri^(2)^.

In their systematic review on drug-induced intracranial hypertension (DIIH), Tan et al.^(3)^ identified that the drugs most strongly associated with DIIH are vitamin A derivatives (especially isotretinoin) and tetracycline-class antibiotics (minocycline, tetracycline, and doxycycline).

One previous observational case series reported 179 cases of intracranial hypertension associated with isotretinoin use, of whom, based on spontaneous reports, 43 (24%) patients had taken tetracycline-class antibiotics beforehand^(4)^.

Physicians should understand the strength of association of each of the aforementioned DIIH medications and exercise caution when prescribing them. A washout period of seven days is recommended when switching between tetracycline-class antibiotics and isotretinoin^(5)^. Physicians should also notice that these seven days are the theoretically calculated seven half-lives needed to achieve 99% drug clearance after the last dose. There is no clinical evidence on the ideal interval, but it is important to stress the danger of associating the two drugs and to understand that because of the rareness of the event, a clinical study on that matter would be hard to perform.
